# Supplementary material for: The root of anomalously specular reflections from solid surfaces on Saturn’s moon Titan
Source: Nat Commun. 2020 Jun 16;11:2829. doi: 10.1038/s41467-020-16663-1 (PMC7298017; doi:10.1038/s41467-020-16663-1)
Supplement: Supplementary file 2 — Description of Additional Supplementary Files [file 41467_2020_16663_MOESM2_ESM.docx]

**Description of Additional Supplementary Files**

**File Name: Supplementary Data 1**

**Description:** Subradar locations of Arecibo Observatory observations using the 2015 International Astronomical Union's spin model.
